# What Can We Do for Amphibians and Reptiles at Schools? Between Personal Conceptions, Conceptual Change and Students’ Pro-Environmental Attitudes

**DOI:** 10.3390/ani9080478

**Published:** 2019-07-24

**Authors:** Zofia Anna Chyleńska, Eliza Rybska

**Affiliations:** Department of Nature Education and Conservation, Adam Mickiewicz University in Poznań, Education, ul. Uniwersytetu Poznańskiego 6, 61-614 Poznań, Poland

**Keywords:** amphibians and reptiles, attitudes, conceptual change, teaching sequence

## Abstract

**Simple Summary:**

Amphibians and reptiles play an important role in ecosystems, usually in the middle of trophic networks, but at the same time they are one of the most endangered groups. Their value seems still to be underestimated by society and is based on many misconceptions and a simple lack of knowledge, which in turn might influence society’s willingness to protect these animals. The authors investigated students’ conceptions about amphibians and reptiles, then designed a teaching sequence in order to trace some possible conceptual changes and to shape pro-environmental attitudes towards amphibians and reptiles.

**Abstract:**

Students’ conceptions and conceptual change are deeply investigated phenomena, and the results of such studies can be implemented in the didactic process. For this research, amphibians and reptiles were chosen, because they are animals which are significant for ecosystems but at the same time are often confused with each other. The goal of this study was to investigate students’ conceptions about both groups, and on the basis of the results to design a teaching sequence (TS) which could lead to conceptual and attitudinal change. Authors used questionnaires and in-depth interviews to investigate conceptions and the results of the intervention. The significant correlations show that students tend to classify animals as amphibians or reptiles according to their skin and their living environment. Students’ conceptions about animals indicate a tendency of being focused on purposefulness towards environment and evolution, and therefore can be considered an essentialist approach. The alternative conceptions did not change much after intervention, and results did not differ between age or gender groups. On the other hand, conceptions after intervention were more developed, and students asked more diverse questions about evolution and environmental protection, indicating their growing interest in these animals and attitudinal change.

## 1. Introduction

One of the aims of a science education is to prepare students for ongoing changes in the world [1]. Our society is also changing, and this includes the amount of time we spend experiencing nature. In 1978 Pyle [1] established the idea of “extinction of experience”, and even back then researchers could partially predict an increasing lack of connection with nature among students. Recently, Soga and Gaston [2] developed this concept and revisited it with a necessary update of literature and presented a new version, of practical use to the scientific community [2]. Two main causes of extinction of experience, according to Soga and Gaston [2], are loss of opportunity and loss of orientation. The first loss is caused typically by the migration of people from rural to urban areas. Because of this change, children have fewer opportunities to observe and interact with nature spontaneously. The other cause is loss of orientation—which is defined as a loss of human positive orientation towards nature—which affects children’s emotions towards, and interactions with nature, and in effect their attitudes to experience and to protect nature. The same problem is noted in the work of Mueller et al. [3], in which they focus on the role of animals in science education. They note that student contact with animals in an out-of-school environment is becoming rarer and that teachers should make opportunities for them to have contact with animals in school, as it might positively influence students’ attitudes towards animals [4,5]. In addition, while conducting these activities, attention should be paid to the ethical aspect, such as shaping a respectful attitude to life [6,7]. Of course, all activities should be carried out with caution, and with respect towards living organisms. Teachers can offer students projects which focus on observing animals during excursions to nature reserves. This kind of interaction is more distant and will not create as strong a bond as direct interaction, although it is likely to negatively affect the welfare of animals. As we are living in a world where the extinction of experience is a commonly observed and spreading phenomenon [3,8], one of the first steps in understanding this process, according to Limón [9] and Bahar [10], is to find out what kind of conceptions and experiences with nature students actually have. 

### 1.1. Amphibians and Reptiles as an Object of Investigation

Amphibians and reptiles are often disliked and/or considered off-putting and constitute one of the most disliked animal groups by children [5,11]. This observation is important, because there is strong link between considering an animal likeable and being willing to protect it [5,11,12,13] In both adults’ and children’s opinion, amphibians are slimy and dangerous as well as disgusting. Both groups of animals are often considered unimportant, and neither they nor their environments deserve to be protected [5,11]. However, in reality, both amphibians and reptiles are among the most valid groups whose well-being is crucial for the fitness of other groups of animals. For example, amphibians are an exceptional taxon in which every adult animal is carnivorous—the only known amphibian herbivores are tadpoles of some species. For this reason, amphibians play a significant role in regulating populations of insects or mollusks, and they are also food for many reptiles, birds and mammals [14]. Moreover, they can serve as indicators of a clean environment. Some amphibians, like the northern crested newt, *Triturus cristatus*, can be considered as “umbrella species”, because, by protecting them, it is possible to protect many other groups of animals [15]. Reptiles also play important role in many ecosystems. They regulate populations of small mammals, amphibians and insects, and their eggs are nutritious food for some mammals. They are considered as typically land animals due to morphological and anatomical features—for example, their reproduction is independent from water thanks to fetal membranes. However, reptiles are endangered because of their threatening look or because of their precious skin, which is used for the production of leather goods. Additionally, a lack of knowledge about them (like species recognition or typical behavior) often leads to animals being killed by accident. For example, in Poland there is only one species of venomous snake—*Viper berus*—but other kinds of snakes like *Natrix natrix* are killed after being mistaken for the viper [16]. Another problem is that amphibians and reptiles are often confused with each other [17,18,19].

### 1.2. Theoretical Background

In science education literature, terms such as conceptions, alternative conceptions and misconceptions are defined in various ways. Some researchers claim that the last two have no place in scientific debate, as all types of conceptions are mental constructs and there is no point in discussing whether they are scientifically correct or not [20,21]. In this article, we use the approach of Gilbert and Watts [22], who describe the term conception as a scientifically correct construct. For the definition of alternative conceptions, we follow Mak et al. [23], who describe them as scientifically incorrect ideas which may have three sources: (i) incorrect subject matter knowledge, (ii) misunderstood or incomplete ideas which children develop, e.g., during classroom lessons, and (iii) informal ideas which arise from common-sense reasoning. The first source of misconceptions is broadly explored in the literature [11,24,25], whereas the second and third causes of misconceptions are not so widely represented. In our previous work, we have highlighted several wrong or misleading statements in textbooks [26]. Furthermore, teachers were found to be prone to making similar inaccuracies [27]. Those mistakes make alternative conceptions even harder to change, as a specific incorrect idea is supported by a source of scientific knowledge. Such a situation serves as an obstacle in the process of conceptual change. By conceptual change we mean the process during which concepts and relationships between them are changed or modified over the course of an individual person’s lifetime [28]. 

Regarding the process of conceptualization and categorization, Rosch [29] proposed that in order to create a scientific concept, first we should help students to create a basic idea, a prototype, and then take it to a superordinate level. Accordingly, if we would like to create the concept of an amphibian, we should first present their most typical representative, such as a frog—which might be presented as prototypical species of the amphibian group—and then present them with more extraordinary and atypical colorful species like salamander. Students frequently categorize organisms on the basis of anatomy, habitat or even name [30]. There are a number of animals whose names suggest that they belong to a different group or species than the one they actually belong to—for example, the horny toad which belongs to *Phrynosoma* is a reptile, or slow worm *Anguis fragilis*, which is also not a worm but a reptile. These kinds of name are part of the culture of different nationalities; part of their folk biology, but they are often the source of alternative conceptions in animal classification by students [30]. 

One possible action, based on that idea, is to design a teaching sequence (TS) which will help students create scientifically correct concepts. A TS [31,32] is a specific tool to make learning meaningful by, for example, achieving an effective conceptual change. According to the TS approach, the first important step is to recognize concepts which arise from sources of scientific knowledge and notice errors or misunderstanding that are present in them. Secondly, after investigating students’ ideas/concepts, researchers should recognize parts of students’ conceptions that might be scientifically incorrect. On such a basis, researchers should design their TS, in which each element of intervention corresponds to revealed alternative conceptions. The next step is to conduct the TS on students, and after that to evaluate the results and to check whether alternative concepts have changed or not [31,32]. On the other hand, many researchers claim, based on psychological essentialism, that this change of alternative conceptions is very hard to achieve because many living or non-living things have an “essence” which describes them and is immutable [33]. This “essence” is often very intuitive and naive, also scientifically incorrect and resistant to change. The essence is prescribed to a specific item, whereas concepts are a much wider idea [34].

One of the major ideas that arise in our mind is that without scientifically correct knowledge about amphibians and reptiles, it would be more difficult to create positive attitudes towards them and ultimately it would be more difficult to protect them [19]. Serpell [35] presented the idea on how our attitudes towards something and its utility might lead to a willingness to protect something, or recognizing it as a threat. In his work he uses babies (as attractive and useful) and spiders (threatening and not useful) as examples. If we adapt this, reptiles appear to be attractive and threatening, and amphibians appear to be unattractive and disgusting [5,11,17]. Sometimes even the color of an animal might positively influence willingness to protect them—animals with asymptotic coloring generate a greater will to protect them than cryptic and inconspicuous ones [36], similarly willingness to protect them is lower if they are threatening and poisonous [37]. The attitude change is an important element which also could be achieved through conceptual change [38]. Some authors claim that it is even easier to achieve it in short classes, as the curricula are overloaded with information [39], and for each class teachers have to help students construct elaborate pieces of new knowledge. Students often experience cognitive overload during classes, especially when they are unable to construct new conceptions [40]. Additionally, what we might observe in school reality is a constant lack of time allowed for usage and processing of new information. Teachers felt that there was insufficient time to develop many interesting and student-centered lesson activities and lesson designs using inquiry-based laboratory work [41] or GIS-based lesson plans [42]. This is also true for simply being able to cover content required by the curriculum [40,43]. Nevertheless, teachers can also work with students at an emotional level to work with their attitudes [4,44]. 

Awareness of these attitudes could be used by teachers as element in their TS, when preparing a lesson about amphibians and reptiles.

### 1.3. Aim and Research Questions 

Taking into consideration the aim of preparing and evaluating a TS in the context of shaping students’ attitudes towards amphibians and reptiles, our goal was divided into smaller sections, includeing: recognizing students’ conceptions about both taxa, examining their scientific sources of knowledge, recognizing alternative conceptions among them, designing a TS, and introducing intervention and evaluating it. Thus, we set out the following research questions:

1. What are the students’ conceptions about amphibians and reptiles?
2. Does the proposed didactic intervention TS support building scientific knowledge (conceptual change) about amphibians and reptiles?
3. Can the proposed didactic intervention change students’ attitudes towards protecting amphibian, reptile and environmental protection?

## 2. Methods

Ten schools participated in this project—five primary schools (PS) and five junior high schools (JHS). From each of them, 40 students were chosen randomly—≈30 who participated in the designed intervention and ≈10 who did not—the control group. Altogether we examined 384 students in the intervention group (from PS age 10–12, and from JHS age 13–15), and 117 in the control group. To validate the research tool, pilot studies were conducted with 120 participants from different schools, then the one chosen for the research project. 

The distribution between age and gender in intervention and control groups did not differ significantly (*p* > 0.05). 

The investigations were carried out following the rules of the Declaration of Helsinki of 1975. The work is in compliance with the applicable ethical regulations for research in Poland.

### 2.1. Research Design 

**Figure 1:**
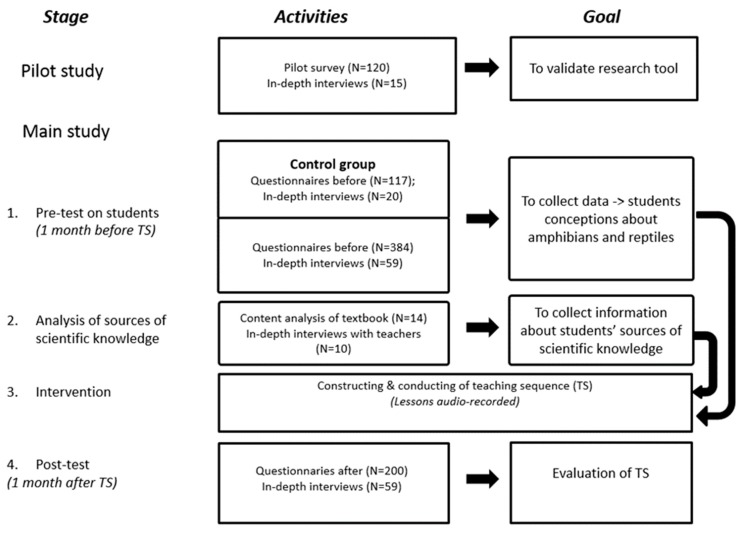
Outline of the conducted project.

In order to find the answers to the research questions, we designed the research with both quantitative and qualitative assessments. Descriptions of the instruments we used are presented in the sections below, and an outline of the project is presented in Figure 1. 

### 2.2. Questionnaires 

Authors provided pre- and post-test surveys, which were constructed after pilot studies with 120 participants. Both questionnaires were the same and consisted of closed, half-open, and open questions concerning amphibians and reptiles and students’ attitudes towards environmental protection [45]. Closed questions were analyzed with quantitative assessment and open with qualitative assessment. 

Pre-test surveys were conducted one month before implementation of TS, and post-test surveys were delivered one month after didactical intervention. The authors created a reference model to which they compared students’ results and assigned points to individual questions. A detailed list of questions and points assigned to the answer can be found in Appendix A.

### 2.3. In-Depth Interviews

Additionally, to receive a deeper understanding of students’ conceptions, in-depth interviews were conducted with randomly chosen students. Based on the pre-test survey, instructions were prepared and consulted on with the help of another researcher. 

Interviews were conducted a month after pre-test and post-test questionnaires. Questions in interviews were designed to have a connection with the questionnaires, to give students the opportunity to elaborate their ideas. Altogether we examined 59 students in in-depth interviews. 

Interviews were transcribed and analyzed with qualitative assessment [46]. Categories used for later analysis were prepared ad hoc during the analysis of the interviews. After analysis of all the interviews, categories with the same meaning were condensed. Inconclusive categories were consulted and discussed by authors, until mutual agreement of how to categorize them was made. A detailed list of questions and categories extracted from the answers can be found in Appendix B.

### 2.4. Designing of the TS 

After analysis of the sources of students’ scientific knowledge [27] and students’ conceptions, a list of common elements in the concepts of “amphibian” and “reptile,” together with alternative conceptions, was made and common elements of those concepts were chosen—a list of them is presented in the results. 

On that basis, the TS was constructed, which was implemented in the schools participating in this study. Hands-on activities were proposed to ensure students’ active participation; a detailed description is presented in Results. All of the proposed activities engaged the whole group, in which they could not only touch presented environments or assign to them matching features but also discuss their ideas with each other using scientific reasoning and debate. The researcher conducting the intervention prompted students when necessary by asking key questions (presented in Results).

### 2.5. Conducting the TS

In order to conduct the lessons in an environment known to the students, and to avoid divergence and relativity in the received data, the lessons were conducted in schools by the same researcher every time (ZC). The researcher asked questions to motivate the students to acquire a deeper understanding of the ideas presented during the lesson. The researcher initially did not explain what the lesson was about and asked the teachers to do the same—to avoid any preemptive learning on the topic, as the authors were aware of teachers’ effect on the studies [47]. 

### 2.6. Analyzing the TS—Audios and Observation

All lessons were audio-recorded; video-recording was not possible because of the lack of agreement from the schools. The researcher extracted the main ideas which appeared in classroom and presented the most interesting dialogues. The questionnaires and in-depth interviews were main measurement tools after the intervention, as described above. Also, to measure results before and after intervention, authors scored tasks in the questionnaires. The maximum number of points was 34. 

### 2.7. Statistical Analysis

All of the data were analyzed with Statistica [48] and R program (R version 3.5.1, Boston, MA, USA). The matrix of correlation was made from the quantified questionnaires in Excel (2013, Microsoft). The statistical significance of the correlation used for the results was 0.05 following the addition of Bonferroni correction.

## 3. Results

### 3.1. Conceptions and Alternative Conceptions Found in Questionnaires’ and Interviews 

#### 3.1.1. Alternative Conceptions Revealed from Surveys and In-Depth Interviews Before Intervention

Both concepts—amphibian and reptile—are quite diverse and extensive. A lot of conceptions and alternative conceptions firstly appeared during the survey and then were developed by students during in-depth interviews. The most significant examples are mentioned below (Table 1 and Table 2).

The conceptions revealed in interviews confirmed the concepts shown in the questionnaires. In this part of the investigation, students were able to give a broader explanation of their ideas. For example, they were able to elaborate why snail and slug, in their opinion, are amphibians, or what elements help them to differentiate between amphibians and reptiles. Examples of a statistically significant correlation are presented in Appendix C.

#### 3.1.2. Correlations between Conceptions and Alternative Conceptions Before Intervention

Some of the conceptions presented above correlate with each other, and some of the most self-explanatory and usable are presented below (Table 3). Students’ conceptions are consistent, even if they are not scientifically correct.

The goal of the questionnaires was to investigate the most common student conceptions about amphibians and reptiles and to investigate how their pro-environmental attitudes towards them are shaped. The results shown in Table 3 present correlations between the opinions presented by students in the questionnaires.

In Table 4 below, correlations between statements found in answers to question 7 in the questionnaire are presented. In this question, researchers asked about student interests in amphibians and reptiles and encouraged them to ask their own questions about these animals. This question was open, and students had various ideas and questions which correlated with various answers from other questions (Table 4).

The statement that students were interested in the protection of reptile or amphibian species correlated with students’ declaration of possessing appropriate knowledge and skills to differentiate and classify given species as amphibians or reptiles, in both cases (r = 0.79). This result might be interpreted as students having appropriate knowledge to recognize which species belong to which taxa; they are also interested in protecting groups of organisms that they know. Some students knew what species of amphibians and reptiles were present in their local environment and stated that they are interested in protecting those animals. Furthermore, statements about interest in protection of the amphibians correlated with the opinion that reptiles’ characteristic movement is slithering (r = 0.67).

Also, questions that students asked about examined taxa of animals indicated that they were interested in the environment in which those animals live. Students often articulated issues which were unclear, for example, whether a snake is a water or land animal, and because of this, they were interested in what environment they live in (r = 0.69). This result might be interpreted as showing that students were aware of their lack of knowledge or misunderstandings about nature and wanted to gain this information. It is worth noting that the students’ confusion is scientifically correct, as reptiles are animals which can live in both a water and a land environment.

The majority of questions considered information about the biggest or smallest representative of amphibians and reptiles:

“What is the biggest amphibian/reptile?”

“Where can I find the biggest amphibian or reptile?”

“How long is the longest snake?”

The second biggest group of questions considered awareness of students’ lack of knowledge:

“How can I differentiate amphibian from a reptile?”

“What is the most important/characteristic feature distinguishing amphibian from a reptile?”

“How long does a frog live? And what does it do for winter?”

Interesting examples of questions asked by students were of a more philosophical nature:

“Why should we protect animals?”

“Why are amphibians and reptiles important?”

“Why should we study (at all) anything about amphibians and reptiles?”

Based on information obtained from surveys and in-depth interviews, researchers designed their TS.

### 3.2. Design of the TS

The TS was constructed based on the results of the surveys and interviews. One of the major goals of the TS was to lead to conceptual change, especially in the area of diagnosed alternative conceptions. Tackled problems and proposed elements of TS are presented in Table 5 below; a full description is presented in Appendix D.

#### 3.2.1. Assessment of the TS Effect

The assessment of the TS was examined by comparing the scores received by students in questionnaires.

Results from the questionnaires presented below appeared in both kinds of schools. There were no differences between students of different ages or gender, as shown by the Mann–Whitney U test. As set out below, differences between results in the intervention group were not statistically significant, although significant difference was noticeable in the control group (Table 6).

The difference in the before and after results between the control group (which had traditional lessons on amphibians and reptiles) and the group participating in the intervention is worth noting.

As presented in the Mann–Whitney U test, groups participating in the intervention had higher scores on the favor of results from questionnaires after intervention, but the result was not statistically significant (Z = −0.66, *p* = 0.5).

Results from the control group were statistically significant, and there was an observable difference between groups (Z = 2.19, *p* = 0.02). The control group had lower results in the post-test survey.

**Figure 2:**
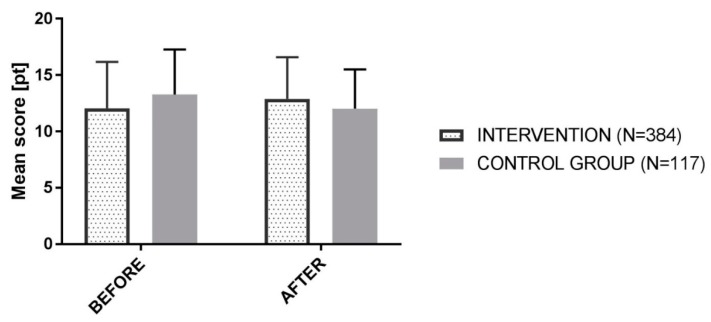
Summary of mean results gained in questionnaires before and after intervention.

In the group from intervention, the results increase by 0.3 points (Before = 12.6, SD = 4.16; After = 12.9, SD = 3.69), and the control group’s results decreased by 1.3 points (Before = 13.27, SD = 4; After = 11.97, SD = 3.48) (Figure 2).

Students, regardless of age, have similar ideas about amphibians and reptiles, and the visualization of the animals in drawings often helps students, but at the same time may lead to misconceptions. Even if students declare to have a good idea as to what species can be classified as amphibian or reptile, when they see photos they often do not know to which group the presented animal should be assigned. For example, they know that the salamander is amphibian, but as it looks like a lizard, they often have a problem with assigning it to the correct group. This conflict also appeared in their dialogues, when they discussed the idea of scales on the skin:

“Student1: Scales belongs to snakes…
Student2: Yes, but fish also have scales…
Student1: But fish live in the water and snakes in the desert.”

Skin was also a debatable idea in the context of the slime on the skin:

“Student1: On the skin of the snail is slime... so it is amphibian?
Student2: I thought it was more like a clam…
Student3: Slime is to keep them moist…
Student2: Maybe slime is more like a universal thing?
Student1: So I think we should assign slime on the skin as an amphibian trait.”

Other morphological element which was confusing for the students were idea of fetal membranes:

“Student1: Amphibians do not have fetal membranes, because they migrate to the ponds to reproduce.
Student2: Yes, but reptiles also do not have fetal membranes.
Student1: But snakes lay eggs on the land...
Student2: Yes, but sea turtles live in the water and also they reproduce in water.”

#### 3.2.2. Students’ Conceptions Revealed from Surveys and In-Depth Interviews After Intervention

During the survey and in-depth interviews, a lot of conceptions and alternative conceptions were documented. The most significant examples are mentioned below in Table 7.

Many of the conceptions were stable and did not change significantly, even though intervention was designed to engage students with hands-on activities. Some of the alternative conceptions have a higher score after intervention, although differences are small and not statistically significant. A lot of conceptions get more orderly distributed, which was also observed in post-test interviews.

Although question 7 considered students’ interest and questions they would like to ask in the post-test interview, there was a bigger variety of questions than expected. Some examples of new questions were the more specific ones about protecting species:

“How we can protect the environments in which amphibians and reptiles live?”

“How we can create a place to protect the animals around our school?”

Also, students had more questions about adaptation and evolution. This concept did not appear at all in questionnaires before:

“How have amphibians evolved?”

“How could amphibians evolve to not be dependent on water?”

Some of the questions revealed an understanding of reptiles as evolutionary more developed than amphibians, but at the same time showed a lack of understanding of the principles of evolution:

“How can a salamander evolve to be a lizard?”

“Is every reptile a relative of the dinosaurs?”

Similar ideas were presented in the post-intervention interviews.

#### 3.2.3. Interviews Post Test

In interviews after intervention, the presented correlation changed, and a new quality of the conception was noted. These conceptions were more elaborate and scientific. The students understood the relationship between the living environment and the morphological and anatomical features of amphibians and reptiles:

“This lizard lives in the desert; it has to have skin with scales so that all of the water will not evaporate.”

In addition, their view on the environment of animal life became more holistic. As in interviews before intervention, students presented as an example of protecting animals’ ideas focused on organism, like protecting the frog by taking care of them, or telling other students to not to kill them. After intervention, in interviews, such statements as the following were made:

“If I know how to distinguish the amphibian from the reptile, I will also know in which environment they live, and we will be able to protect this place. If we had a place near the school, we would make a reserve there, and we would not be able to enter it.”

Alternative conceptions, which rarely appear again after intervention, were a snail or slug as an amphibian. A new conception that appeared was an evolutionary reference in the context of differences between amphibians and reptiles, and also in the context of particular evolutionary advantages, like fetal membranes, developed lungs, and reproduction independent from water:

“Snakes bury their eggs in the sand, because their eggs have protection membranes. Amphibians do not have that, so they have to lay eggs in the water.”

As in the surveys, questions arose about the evolution of how amphibians and reptiles adapt to their environment:

“Can amphibians adapt themselves to a land environment like reptiles?”

“How long did the evolution of the frog take?”

Also, in interviews after intervention, statistically significant correlations between statements about the environment and evolutionary advantages were observed.

## 4. Discussion

The major goal of the study was to design a TS which will allow for a conceptual change regarding amphibians and reptiles. However, our TS more greatly impacted a broad spectrum than only knowledge about categorization. Designed intervention did change students’ conceptions, although in a smaller range than we expected; the majority of conceptions and alternative conceptions remained constant in the questionnaires. The in-depth interviews show a stronger effect of conceptual change. There was no difference in those results between age or gender groups. The TS was conducted in the timeframe which is normally allotted to this type of class about amphibians and reptiles, so it could be assumed that the results are comparative. The most interesting result was the change in students’ attitudes towards amphibians and reptiles being more pro-environmental. Authors of this publication claim that this might be attributable to the reasons discussed below.

One possible explanation of this result lies in the problems associated with understanding the concept of animal classification. Classification as an idea is complicated and artificial, which makes it very hard to teach and learn. Regardless of the group of animals, alternative conceptions about different taxa are quite common. Even the sole concept of animal classification is hard to follow and understand [49]. Also as earlier mentioned in the article, society does not tend to protect something which they do not understand. Hence, if they would not be able to differentiate amphibians from reptiles, there is a lower chance that they will tend to protect both of these groups.

The results could also be influenced by attitudes towards amphibians and reptiles, which are mainly negative. Tomažič and Sorgo [5] investigated and showed negative attitudes, for example, towards toads. In earlier research by Prokop and Tunnicliffe [11], amphibians and reptiles are presented as disgusting animals. Our research indicates that students have shown increasing interest in protecting these animals. Our results indicate that gaining personal experience with animals which might appear as disgusting, leads to building personal knowledge about them, understanding connection between particular features and environment (e.g., slimy skin as adaptation allowing for gas exchange in amphibians) and bigger acceptance of it. All mentioned steps results in a change in students’ perception and those animals might be not so disgusting as they appear at the first place. Thus, it could be an indicator of changing attitude, and changing an attitude is an important step in creating intrinsic motivation for learning, and can lead to students wish to protect it.

### 4.1. Answers to Research Questions

The goal of the first research question was to investigate students’ conceptions about amphibians and reptiles. The results were more descriptive, and information from the research questions enabled us to construct a TS. As conceptions resulting from students’ answers were also supplemented by information from sources of scientific knowledge, the authors had almost complete information for how to design the most suitable TS.

The first major conceptions which were found during the pre-test were predictable, for example, salamander as a lizard. Another misconception, which was not reported in the literature, involved classifying slugs and snails as amphibians. What is even more interesting is that this alternative conception was resistant to change for some students. However, some publications show similar misconceptions [5,11,19,50] which, together with the data presented here, can be used to explain students’ reasoning behind it. For example, mollusks were classified as amphibians because of their movement (near the ground and slow) and skin attributes (smooth and slimy). As presented in the work of Chyleńska and Rybska [30], visible similarities were key to classification, and that is why slugs and snails might be classified as amphibians. Similar results can be observed in the work of Yen et al. [19], where crickets were classified as reptiles because they live in warm fields, appear mostly on sunny days, and move very quickly. Also, Crump and Fenolio [17] note that alternative conceptions, especially those deeply rooted in heritage and folk biology, might be resistant to change. For example, the characteristic word to describe a snake is slimy, which might lead to alternative conceptions about snakeskin that appeared in our results before and after intervention. In this research students were also deeply convinced that snails and slugs are amphibians, but spiders, which theoretically might be mistakenly connected with reptiles, were assigned to the “other animals” category without hesitation. That difference might be explained by the findings of Serpell [35], where spiders have been recognized as relevant to human interest (as they might be dangerous) but also as scary/frightening—the same can be said about snakes, and mollusks appear in other quarters where we loathe them but there is no interest in them—as with amphibians. At some point it does not matter how an animal looks or is built—the texture of the skin is used here to differentiate groups, and snails fit in perfectly as ones with slimy skin considered as disgusting, as is the case for amphibians—both groups are in the same perceptions category [17,35]. However, the lack of statistically significant change in that misconception after the TS might be explained by the fact that the presented intervention was designed neither to discuss a general classification idea nor to cover topics related to snails. In fact, the word snail did not appear during the intervention. What is also worth mentioning is that, in the Polish language, words which refer to vipers or reptiles are considered offensive when used to describe a person as sneaky or vile, and it does not help in shaping more positive attitudes towards them.

### 4.2. Effect of TS—Attitude Change

Intervention did not eliminate existing alternative conceptions. It shifted them, but permanent change requires more factors. An important finding is that conceptions did not differ between age groups and gender, neither before nor after intervention. It is consistent with some results known from the literature, where students also had similar alternative concepts, regardless of age or gender, e.g., Yen et al. [19], but there were no didactical interventions conducted to change these concepts.

Moreover, what we would like to highlight here is that our intervention stimulated a rise of students’ interest in and reflection on those animals. It was observed that after intervention they refer to more elaborate stories, concerning more encounters which they had or more importantly how they would change the neighborhood around their school to make it more accommodating to amphibians and reptiles. Additionally, when students were describing these changes, they had a clear idea in which environment amphibians and reptiles can be observed. This outcome corresponds with the recent work of Tomažič et al. [37] which found that direct contact even with animals which provoke negative attitudes, such as venomous and poisonous animals, can lead to a change of attitude. As presented in this article, intervention might not lead to significant conceptual change but certainly leads to a change of attitudes, which is equally important. As shown in a study by Rule and Harrell [51], such a change might be an outcome of designed teaching sequences. Moreover, it is consistent with other studies by these authors where it is shown that attitudes are crucial to conceptual change, and if they are improved and changed to be more pro-environmental, then change of conceptions will follow [11,52,53].

Interestingly, a statistically significant difference before and after intervention was (also) found in the control group. The results of the control group decreased over time. It shows that, even though improved results from the intervention group were not statistically significant, the intervention might influence the students in the end. The decreasing results of the control group agree with many publications which indicate that classes that do not involve students in the didactic process, or stimulate them to create new thinking constructs, may have a smaller impact on the students, and the results may be less durable. At the same time, the control group more often correctly classified salamander as an amphibian when compared to post-intervention students, despite overall worse results with classifying other animals. This might be explained by results from textbook analysis where salamanders were found to be widely represented as amphibians because of their attractive appearance, in great disproportion to newts—another representative of Caudata amphibians [27]. Also, this conception is consistent with the results of intervention, where students correctly classified in post-test salamanders as amphibians, but by guessing and not by reflective analysis. On the contrary, when students were asked to think about features of the salamander, in some cases they backed out from their initial answer, and classified it as a reptile, even claiming that they have scales, as reptiles have. Moreover, in other cases, during in-depth interviews students who assigned salamander to amphibians became confused and changed their answer when they were shown the photo of the species. If we consider the skin and environment as crucial factors in students’ reasoning, we can observe that they are very direct in their thinking—they tend to follow Lamarckian logic in their thinking about evolution. It appears that they are not able to acquire evolutionary concepts and see that amphibians’ and reptiles’ adaptation to the environment is a direct correlation, i.e., not random. This is connected with the results of Fiedler et al. [54], who suggest that evolution as a whole concept might be hard for students to understand, due to misunderstanding concepts like randomness or probability. Also, the essence of the evolution (according to the theory of psychological essentialism) is different to facts which result from scientific knowledge. Therefore, students’ alternative conceptions, which are intuitive, might be resistant to change [55].

Accordingly, important factors used to differentiate discussed taxa are method of reproduction and adaptation to environment connected with it, which incidentally appeared in students’ responses. That said, one feature which was very easy for students to assign to the correct taxa was the construction of an egg. Students intuitively knew which eggs were adapted to which environment and were able to assign fetal membranes to the appropriate environment, but they seemed not to understand that this was a result of evolution. Similar results appear in work of other authors, which suggests that students have a problem with counter-intuitive explanation of evolutionary phenomena [56].

In any case, the research itself and the intervention were aimed not only at teaching students to classify but also at changing their approach to animals to be more scientific. Researchers planned to change the concept at a higher level of understanding of the natural world and its principles, so that students would be able to apply this skill more extensively, and that this would accordingly affect their pro-environmental attitudes. In this case, we have achieved success through our intervention, and we made a conceptual change. As presented in the results of the conducted interviews, after the intervention, many students were interested in the protection of the amphibians’ and reptiles’ environment; they had specific ideas and solutions, and were aware that if we want to protect animals, we cannot look only at individual species, but also at their surroundings. More importantly, as it appears from our results, besides teaching about the classification of animals and the environment, teachers and educators should also put a focus on the life cycle of those animals, which shows a crucial difference between amphibians and reptiles. By simple showing students during reproductive season a pond with amphibians eggs in it, we can elaborate on crucial differences between amphibians and reptiles. Through providing students with the opportunity to experience the animals, we gave them the chance to interact with and observe them. It also created a space for orientation [57,58]. This may indicate that introducing live animals into the classroom may not always change students’ conceptions but is very promising in changing their experiences and helps to reduce the “extinction of experience” effect by creating a space for student “opportunities” and “orientation” needed in modern society. In particular, the modern curriculum does not encourage teachers to allow for students’ contact with living animals and excursions to their natural environment, and the number of lessons for biology was reduced; thus, the ongoing situation requires a view on the curriculum and reshaping it to encourage students to do so. Preferably, as an action, taking students on field trips and observing animals in their natural environment is best for the animals’ welfare. On the other hand, keeping animals alive in a classroom as pets with all the ethical aspects preserved may have better additional results, such as shaping the attitude of responsibility, observing them regularly etc. This could presumably be more influential than a one-day excursion.

## 5. Conclusions

Our research shows that students have different ideas about the taxa of amphibians and reptiles, but also that attitudes towards them might be an underestimated element in helping students to develop scientifically correct knowledge. What we have shown in our research is that, regardless of age, gender, or school, students had very similar conceptions, the majority of which resist change even after conducting the designed TS with the inclusion of student-activating hands-on tasks. The strongest argument that arises from our current research is that introducing live animals into the classroom might help not only in reducing the effect of “extinction of experience”, but also in changing students’ attitudes towards animals, and broadening their perception of these animals. We conclude that this is our most important outcome.

We have shown also that students know that there are some differences between amphibians and reptiles, although they have a problem in assigning particular features or labels to particular taxa. We have shown that there are specific features which a teacher should focus on while teaching about examined taxa, such as skin and reproduction in the context of the living environment and evolution. This is especially the case because students’ thinking about adaptation and evolution is direct and they are essentialist in their way of thinking about these processes.

## Figures and Tables

**Table 1:** Examples of conceptions which arise from answers before intervention about amphibians.

**Most Common Student Conceptions about Amphibians**	
1.	Amphibian is something small, moves slowly; may walk or jump or slither; their skin is moist with mucus. Living environment might be land or water-land.
2.	Frog is an amphibian because it reproduces in water, has slimy and moist skin, abdominal flattening, amphibious living environment, slow movement—jumping or walking.
3.	Salamander is an amphibian because of its movement, skin features.
**Most Common Student Alternative Conceptions about Amphibians**	
4.	Slug and the Roman snail are amphibians because of their movement; slime as characteristic of an amphibian.
5.	Snake is an amphibian but still has scales, specific movement—leaning, swimming, or wriggling—which differentiates snakes from other group of animals.

**Table 2:** Examples of conceptions which arise from answers before intervention about reptiles.

**Most Common Student Conceptions about Reptiles**	
1.	Reptile has dry skin with scales, lives in the desert or any dry environment, lays eggs, usually possesses limbs (except for snakes) so it can walk and run, possesses a tail.
2.	Snake is a reptile because of its dry skin and scales, abdominal flattening, living environment, crawling as a movement, skin is correctly correlated with being dry; scales. Also some students claimed that it is a reptile due to a lack of limbs.
3.	Lizard is a reptile because of its living environment and own information of students—their own experiences, as lizards are quite common and easy to observe; they may walk or crawl, but there is no correlation with the skin.
**Most Common Student Alternative Conception about Reptiles**	
4.	Salamander is a reptile because of its living environment (on land) or morphological similarity to lizards (legs and tail).

**Table 3:** Correlation between conceptions and alternative conceptions of amphibians and reptiles from questionnaires.

Statement	Correlated with Statements	Strength of Correlation (r)
A characteristic way of moving for reptiles is using limbs	Reptiles have scales on their skin A characteristic way of amphibians moving is slithering	0.7
Reptiles have scales on their skin	Amphibians have slimy skin	0.6
The feature distinguishing the amphibian from the reptile is their living environment	Amphibians move by jumping	0.6
	Amphibians move by slithering	0.7
Amphibians move by jumping	Reptiles move by walking	0.6

**Table 4:** Correlation between concepts and alternative conceptions about amphibians and reptiles from question 7 of the questionnaire in connection with answers to other questions.

Statement	Correlated with Statements	Strength of Correlation (r)
I am interested in protection of amphibian/reptile species	I know which species are amphibians or reptiles from my own interest in nature	0.79
	Reptiles move by slithering	0.67
Snakes live in water-land environment	I am interested in the living environment of amphibians/reptiles	0.69

**Table 5:** Tasks designed for teaching sequence, on the base of conception or area of interest to which it relates and key questions which were asked during conducting the classes.

Task	Description of Task	Conception or Area of Interest to Which It Relates	Key Question
1.	Students have ability to touch different environments, imagine and suggest what animals may live there (1—moist earth, water and moss, 2—dry rocks, barks, sticks) Biological goal: present a broad perspective/idea that environment shapes organisms Pedagogical goal: activate students—hands-on activity	Environment of living—students assign water environment to reptiles and land environment to amphibians	What kind of animal fits each environment (dry vs. wet)?
2.	Students assign chosen animal traits to environments Justification of their choice Biological goal: introducing evolutionary approach Pedagogical goal: collaborative learning, discussion—collaborative meaning making	Adaptation to environment: lack of knowledge about elements of amphibian and reptile morphology	What features might be helpful/needed in order to live there?
3.	Constructing models of a skin Biological goal: introducing evolutionary approach using the example of skin Pedagogical goal: introducing the idea of a simple scientific model into teaching, scientific reasoning with the usage of model	Students differentiate skin of amphibians and reptiles but cannot ascribe it to adaptation to the typical environment	What kind of skin better suits a particular environment? What skin features help animals to survive in water/land environment?
4.	Assigning representatives of native amphibians and reptiles to each taxa Biological goal: introducing the idea of classification and connection between morphological representatives Pedagogical goal: collaborative decision-making	Students assign illustrations of amphibian and reptile representatives to chosen features and environments	What are the features of this representative that make you assign it to this environment?
5.	Possibility to meet living representatives of both clusters during classes Biological goal: experiencing live animals, shaping observation skills Pedagogical goal: consolidation of information and experience	Classification—lack of connection between theoretical knowledge about amphibians and reptiles and living representatives of amphibians and reptiles	What amphibians and reptiles did you meet in your life? What interesting things do you notice about bearded dragon and *Bombina* species.

**Table 6:** Summary of Mann–Whitney U test from results of questionnaires before and after intervention.

Group	Mann–Whitney U test, *p* < 0.05						
	Sum. Rang	Sum. Rang	U	Z	*P*	*N*-Bef.	*N*-Aft.
Intervention	106,342.0	50,178.00	32,422.00	–0.664925	0.506099	384	200
control group	12,974.00	8141.000	4225.000	2.194265	0.028217	117	88

*N*-Bef.: number of students before intervention; *N*-Aft.: number of students after intervention.

**Table 7:** Examples of conceptions and alternative conceptions which arise from questionnaires after intervention.

**Conceptions and Alternative Conceptions about Amphibians**		**% Before**	**% After**
1.	Salamander is an amphibian, living environment—land (L), or both environments—water and land (WL), the movement is walking, wet skin.	42 (L) 69 (WL) 25 7 9	47 (L) 70 (WL) 26 6 19
2.	Frog is an amphibian because of slimy moist skin which sometimes might be rough, also because of: abdominal flattening, amphibious environment of living, slow movement—jumping, walking.	81	80
3.	Toad is an amphibian—skin (plain and wet, sometimes considered rough), environment of living, jumping, crawling as a movement.	79	77
4.	Slug (S) and Roman snail (RS) are amphibians.	(S) 21 (RS) 17	22 20
5.	Snake is an amphibian because of dry, rough skin and living environment.	19	23
**Conceptions and Alternative Conceptions about Reptiles**			
1.	Snake is a reptile because of skin, abdominal flattening, living environment, crawling as a movement, skin is correctly correlated with being dry, scales.	73	69
2.	Lizard is a reptile because of living environment and own information of students—their own experiences.	80	80
3.	Turtle is a reptile because of how it is built, skin which is hard and dry, way of movement.	56	56
4.	Salamander is a reptile as it may have scales, and students guess that they are reptiles—no experience with this kind of animal migh be a result of their knowledge about them.	53	50
5.	Toad as a reptile has rough skin.	14	15

## Appendix A

### Questionnaire

1. Look at the pictures and:
  (in questionnarie below pictures of the following species were used. For those illustration we had a right to use them for the research itself, but we did not have the rights to use them in this publication)
  A. slug (*Limulus maximus*)
  B. viper(*Vipera berus*)
  C.salamander (*Salmandra salamandra*)
  D. spider (*Eratigena atrica*)
  E. fish (*Cyprinus carpio*)
  F.lizard (*Varanus panoptes*)
  G. frog (*Rana temporaria*)
  H. snail (*Helix pomatia*)
  I. toad (*Bufo bufo*)
  J. turtle (*Terrapene carolina*)
  (A) Group animals to amphibians, reptiles or other (10 points)
  (B) Explain what was the reason to assign animals to each group. (3points)
  (C) Assign each picture to environment of living: water, water-land or land environment (10 points).
2. Compare how the amphibians and reptiles move (2 points)
3. Would you feel the difference between skin of amphibian and retile—if yes describe it (3 points)
4. Imagine that you went on the trip with a friend. You meet an animal and you claim that animal is an amphibian and your friends says that is a reptile. How you will decide which of you is right? (2 points)
5. Which of mentioned species are the amphibians—choose one answer and explain your choice (2 points)
6. Which of mentioned species are the reptiles—choose one answer and explain your choice (2 points)
7. A—Ask your own question about amphibians
  B—Ask your own question about reptiles

## Appendix B

**Table A1:** In-depth interviews with students.

Question	Categories
1. What do you think distinguishes amphibian from reptile? The leader asks you to list features to help ask question 3 - projection.	Dry skin; Moist skin Limbs present/Lack of limbs
2. Imagine that during the holidays, you were on a trip with your sister and cousin. Your sister thinks it’s a reptile, while your cousin thinks it’s a amphibian. What will allow you to finally decide which taxon the animal belongs to—what are the features, will you know if the animal is a reptile or a reptile?	Movement Enviornemnt of living Reproduction
3. Compare the way amphibians and reptiles move.	Reptiles slither, amphibians jump
4. Would touching the skin of the amphibian and skin of the reptile feel the difference? If so, which one?	Amphibian is moist, reptile dry or moist
5. Name two representatives of amphibians and reptiles. Why do you think they belong to this taxon?	Amphibian—frog, reptile—snake, lizard
6. Now I will present you some pictures of the animals—decide to which taxon they should be assigned to amphibians reptiles or the other group (in interviews photos and drawings of the following species were used. For those illustration we had a right to use them for the research itself, but we did not have the rights to use them in this publication:	Proper assign to category: amphibians, reptiles or other
a. Newt (*Triturus cristatus*) in the water environment in the background	
b. Snail (*Helix pomatia*) in natural environment in the background	
c. Drawing of the snail (*Helix pomatia*) with the no background	-
d. Slug (*Limax maximus*) in natural environment	
e. Lizard (*Lacerta agilis*) in natural environment	
f. Salamander (*Salmandra salamandra*) photo in natural environment	
g. Slamander (*Salmandra salamandra*) drawing with no background	
h. Drawing of the lizard (*Lacerta agilis*) with no background.	
i. Drawing of the newt (*Triturus cristatus*) with no background	
7. Would you like ask any question to me (interviewer)?	

## Appendix C

### Description of Correlations before Intervention

**Table A2:** Correlations between statements which appeared in in-depth interviews before intervention. (*n* = 59).

Statement	Correlated with Statements	Strength of Correlation (r)
Amphibians skin is wet and reptiles skin is dry	Amphibians live on land and reptiles in water	−0.44
	Elements to differentiate amphibian from a reptile are skin (amphibian is slippery and reptiles has scales), way of respiration (amphibian larva has gills, reptiles have only lungs and no larva), and legs (amphibians have legs and reptiles don’t)	0.33
	Salamander is an amphibian on the photo	0.26
	Triton is reptile on the drawing	0.34
	Lizard is reptile on the photography	0.31
	Lizard is the reptile on the drawing	0.38
Amphibians have legs and reptiles don’t	Reptiles walk and amphibians crawl	0.7
Reproduction as an element to differentiate these two taxa - amphibians dependent on the water, reptiles are independent from water, as they have fetal membranes	Scales, respiration and legs as elements to differentiate amphibian from a reptile	0.43
Amphibians are slippery	Salamander recognized as amphibian on the photo in natural environment	0.53
Environment as an element to differentiate these two taxa	Salamander as an amphibian	−0.26
	Frog lives in water and lizard on land	0.27
	Lizard is a reptile	0.33
	Salamander is a reptile	0.27
Amphibians are evolutionary older than reptiles	Frog lives in water and lizard on land	0.34

There are strong correlations between diagnosed opinions. For example, in students’ opinion, reptiles are moving with a help of limbs and this statement correlates with opinion that characteristic element of reptiles skin are scales (r = 0.7). Furthermore, opinions that characteristic element for reptiles skin are scales, correlates with statements that characteristic element for amphibians skin is a slime (r = 0.6).

Both of this observation might indicate that students are able to observe and notice morphological features and connect them with appropriate taxa.

Likewise, students’ answers presented their awareness about movement of those animals. Thus, statements about amphibians slithering or jumping correlate with their aquatic environment of living (slithering, r = 0.7; jumping, r = 0.6). Moreover the belief that amphibians jumps, correlates with the statement that characteristic way of movement for reptiles is walking (r = 0.6). Those observations indicate that students are able to connect kind of movement with environment, and also assign it to appropriate taxa.

## Appendix D

### TS—Description of Intervention and List of Features

At the beginning of the intervention students were divided into groups and each group received two boxes. In one of them there was wet soil, with moss and in the other dry sand with some rocks and dry sticks and pieces of wood bark. Researcher asked students to close eyes and touch both environments. After that each group had to describe what they felt. Researcher asked them what kind of animals they would expect in each of those two environments and based on what they choose specifically those animals – and furthermore what features they might need in order to live there?

Task 2. In the next phase groups of students receive envelope with examples of different morphological features written on pieces of paper (e.g., different kinds of: limbs—with claws or rebate in the end; kinds of skin—dry, with scales or with mucus; kinds of eyelids etc. —full list of features is presented below). Students had a task to match those received features with the environments from the first part of the lesson, and students had to discuss their choices in the groups until they agree unanimously. Next stage of the sequence involved debate in the class forum. Each group had to read one feature out loud and voice theirs group choice of environment and the rest of the class had to agree or disagree with this decision—and justify their point of view. The debate lasted until all features were discussed and matched with the appropriate environment.

At this point of the sequence the researcher asked groups whether they could guess what groups of animals is this subject of the lesson. Next, the groups received exemplified models of the two kinds of the skin—one which was wet and smooth, and the other which was dry and rough—accordingly students were asked to match presented types of skin to the discussed environments (Task 3). After that, students received illustrations of the representatives of both amphibians—*Triton vulgaris* and reptiles *Lacerta agilis*—and they were asked to match them to the environment for the last time (Task 4). The final stage of the intervention was to present the students with living examples of both animal groups to allow them to observe described features and adaptation to the environments. Fire bellied toad (*Bombina orientalis)* and bearded dragon *(Pogona vitticeps)* were chosen as a representatives of amphibians was and reptiles (Task 5).

List of features:

**Table A3:** List of features.

Amphibians	Reptiles
Limbs with webbed feet	Limbs ending with claws
Limbs with rebates	
Skin covered with mucus	Dry skin
Smooth skin, no scales (naked)	Skin covered with scales
The upper eyelid is immovable, the lower eyelid is transparent	The eyelids are movable, not transparent, of some species fused and transparent
Eyes that respond only to movement	Eyes responsive to moving and immovable objects
Streamlined body shape	The oblong shape of the body
Eggs layed in water environment	Eggs layed in a terrestrial environment
Eggs in a jelly-like, transparent shell	Eggs in an not transparent casing
Lack of fetal membranes.	Fetal membranes present
Limbs (if any) on the sides of the body	Limb’s body (if any) lift the body above the ground
There are numerous glands in the skin.	There are no glands in the skin.
No head mobility.	Mobility of the head in different directions

As 10 classes were attending to intervention, there were 4 groups in each class; the maximum amount of points to gain in measured Task 2 was 40.

During the part of the lesson where students were asked to assign particular morphological elements to one of the environments we counted the number of traits correctly assigned to each group/environment.

Students most often assigned four out of eleven factors connected with amphibians and three out of twelve for reptiles. Specific results are presented in Table A4 below. Detailed description is presented.

**Table A4:** Factors which students assigned as typical to amphibian or reptile in their environment during Task 2 (maximum points 40).

Amphibians	Number of Points	Reptiles	Number of Points
Limbs with swimming membranes	10	limbs with claws	40
skin covered in slime	40	dry skin	40
smooth skin without scales	40	skin with scales	22
upper eyelid, non-transparent, lower eyelid, still and transparent	3	The eyelids are movable, opaque, of some species fused and transparent.	27
eyes reacting only on movement	10	eyes reacting both on moving and non-moving objects	3
Streamlined body shape	35	an elongated body shape	22
eggs lays in water environment	40	eggs are laid on the land	40
eggs in a jelly-like, transparent shell	40	eggs in nontransparent thick shell	35
lack of fetal membranes	3	fetal membranes are present	18
limbs (if they are) on the sides of the body	5	limbs (if they are) are lifting the body	3
there are numerous glands in the skin	31	there are no glands in the skin	31
lack of head mobility	27	head with increased mobility	3

We found out that some traits were for students much easier to assign then others and for example: skin covered with slime and eggs in jelly-like, transparent shell, which are laid in water environment were correctly assigned to amphibians (by 40/40 of groups participating in the TS). Streamlined body shape was matched with wet environment by 35 out of 40 groups, and so were numerous glands in the skin (31/40) and lack of head mobility (27/40). For reptiles, the most correctly assigned by students’ traits were: limbs with claws, dry skin, and eggs laid on the land (assigned to reptiles by 40 out of 40 groups each)**.** In almost every school, to the environment of reptiles students assigned: eggs in nontransparent membrane (assigned to reptiles by 10 out of 40 groups), there are no gland in the skin (assigned to reptiles by 30 out of 40 groups), or the eyelids which are movable, opaque, of some species fused and transparent (assigned to reptiles by 27 out of 40 groups). Interesting are those features which were correctly assigned in all groups—so high correctness might have connection with hands-on activity in which students were involved. Touching of two different environments and collaboration groups helped students to appropriately assign features which were adaptation suitable to environments.
